# Autonomic Function in Parkinson's Disease Subjects Across Repeated Short-Term Dry Immersion: Evidence From Linear and Non-linear HRV Parameters

**DOI:** 10.3389/fphys.2021.712365

**Published:** 2021-10-06

**Authors:** Liudmila Gerasimova-Meigal, Alexander Meigal, Nadezhda Sireneva, Irina Saenko

**Affiliations:** ^1^Department of Physiology and Pathophysiology, Petrozavodsk State University, Petrozavodsk, Russia; ^2^Institute of Biomedical Problems, Russian Academy of Sciences (RAS), Moscow, Russia

**Keywords:** dry immersion (DI), Parkinson's disease (PD), heart rate variabiity (HRV), blood pressure (BP), heart rate (HR)

## Abstract

Several studies have shown that “dry” immersion appears as a promising method of rehabilitation for Parkinson's disease. Still, little is known about the cardiovascular reaction in “dry” immersion (DI), especially in Parkinson's disease (PD). Therefore, this study was aimed to evaluate the effect of repeated 45-min DI sessions on autonomic function in subjects with PD. The study group consisted of 20 subjects with PD [13 men, seven women, aged 51–66 years old, Hoehn & Yahr (H&Y) staged 1–3] were enrolled in the study according to inclusion and non-inclusion criteria. The DI program was comprised of seven 45-min DI sessions, applied within 25–30 days. Blood pressure (BP), heart rate (HR), and electrocardiogram (ECG) in the standard lead II were recorded at 1st, 4th, and 7th DI, before, on the 15, 30, and 40th min of DI session. Autonomic function was assessed with analysis of heart rate variability (HRV) using Kubios Standard version 2 software. Linear (time- and frequency-domain) and non-linear (correlation dimension, entropies, DFA1 and DFA2, percent of determinism, and recurrence) were computed. At baseline condition, time- and frequency-domain HRV parameters showed low variability of HR, which indicates reduced autonomic neurogenic control of HR. Throughout the DI session, systolic and diastolic BP has decreased by 5–7 mm Hg (*p* < 0.001), and time- and frequency-domain parameters of HRV have significantly increased, what can be regarded as compensatory mechanisms of hemodynamics during DI. The structure of the regulatory input to the heart seen by HRV was characterized by low complexity and reduced autonomic neurogenic control of HR. Across the program of DI sessions, the hypotensive effect was documented, but no notable modification of the HRV-parameters was found. The absence of long-term modification of the studied parameters can be attributed both to deconditioning environmental effect of DI and limited adaptation of the organism due to neurodegeneration in PD. That should be taken into consideration when planning rehabilitation measures in subjects of older age and chronic somatic diseases with modeled microgravity.

## Introduction

“Dry” immersion, along with antiorthostatic hypokinesia and parabolic flights, refers to methods of Earth-based modeling of microgravity (Pandiarajan and Hargens, 2020). Under modeled microgravity, the body of the subject experiences the main effects of space flight, such as redistribution of the extracellular fluid of the body, hypokinesia, and lack of support (Tomilovskaya et al., 2019; Pandiarajan and Hargens, 2020). Compared with other methods, “dry” immersion (DI) is considered the most sparing method of modeling microgravity in Earth conditions, since the redistribution of blood to the upper body and head that occurs during DI session is not so pronounced as with other methods, e.g., antiorthostatic hypokinesia (Watenpaugh, 2016; Tomilovskaya et al., 2019; Amirova et al., 2020).

Besides space physiology, DI has begun to be used in rehabilitation to normalize increased muscle tone and blood pressure (BP) in patients with chronic diseases of the nervous, circulatory, and musculoskeletal systems (Tomilovskaya et al., 2019).

Earlier we have shown that ground-based microgravity modeled with a short-term session of DI diminished tremor and muscle rigidity in Parkinson's disease (PD; Meigal et al., 2018, 2021a; Miroshnichenko et al., 2018), which can be attributed to the well-established atonia-inducing effect of DI on the skeletal muscle of healthy subjects (Navasiolava et al., 2011; Amirova et al., 2020). Additionally, the performance of the visual-motor reaction time tasks with higher cognition demand has improved after a program of DI sessions (Meigal et al., 2021b). However, these positive effects do not translate to quality of life and activity of daily living, or body balance (Meigal et al., 2018, 2021a).

Parkinson's disease is known as neurodegenerative disease, characterized by lowered dopamine production in the central nervous system (CNS) what leads to several cardinal motor symptoms, such as rest tremor, elevated muscle tone or rigidity, brady- or akinesia, and impairment of postural reactivity and gait (Rodriguez-Oroz et al., 2009; Reich and Savitt, 2019). Besides motor and cognition deficits, dysfunction of the autonomic nervous system, or dysautonomia, is a common feature of PD (Metzger and Emborg, 2019), which often precedes the motor symptoms (Chen et al., 2020). Dysautonomia in PD is represented by cardiovascular, gastrointestinal, and urinary disorders, hyperhidrosis, sexual dysfunction, thermoregulatory aberrance, and pupillo-motor abnormalities (Postuma et al., 2013; Metzger and Emborg, 2019; Chen et al., 2020). Autonomic dysregulation in PD is determined by neurodegeneration associated with alpha-synuclein deposition in neurons of the central and peripheral autonomic nervous system (Chen et al., 2020), including the enteral nervous system (Jain, 2011). There are studies that allowed hypothesizing that the “gut-brain axis” also contributes to PD pathogenesis (Schaeffer et al., 2020; Lee et al., 2021). Cardiac and extra-cardiac sympathetic denervation, provoked by alpha-synuclein deposition in autonomic nerves, along with arterial baroreflex failure is regarded as major mechanisms for orthostatic hypotension, BP lability, and supine hypertension (Jain and Goldstein, 2012). Signs of orthostatic hypotension are seen in 40% of subjects with advanced PD (Jain and Goldstein, 2012).

Heart rate variability in PD is vastly investigated with traditional linear parameters, which indicate diminished sympathetic and parasympathetic autonomic activity (Jain, 2011; Jain and Goldstein, 2012; Soares et al., 2013; Maetzler et al., 2015; Gibbons et al., 2017; Palma and Kaufmann, 2018; Akbilgic et al., 2020; Li et al., 2020). Only a few studies on non-linear parameters of heart rate variability (HRV) in PD are available in pre-existing literature (Kallio et al., 2002; Pursiainen et al., 2002; Palma and Kaufmann, 2018), though non-linear parameters of HRV are widely used to study other kinds of pathologies (Voss et al., 2009; Shaffer and Ginsberg, 2017). Non-linear parameters inform the temporal structure and complex patterns of a signal, and they often outmatch the linear ones by sensitivity to specific features of biosignals. For example, entropy, correlation dimension, and rate of recurrence of surface electromyogram presented better sensitivity to clinical motor symptoms in subjects with PD (Meigal et al., 2013).

In our earlier study in subjects with PD, we found that BP has modestly decreased after a single 45-min session of DI (Meigal et al., 2018). Also, the autonomic dysfunction, evaluated with a rating scale, has decreased by 50% after a program of seven sessions of DI (Meigal et al., 2017, 2020). Similarly, the modest decrease of BP and heart rate (HR), as well as the increased variability of HR, which evidenced on compensatory mechanisms, was detected during 45-min DI sessions in healthy young subjects (Gerasimova-Meigal and Meigal, 2019; Meigal et al., 2020). These data are in line with the dynamics of BP and HR in healthy young subjects during short-term DI (3–5 days; Tomilovskaya et al., 2019; Amirova et al., 2020). Nonetheless, many humoral factors, which are important for systemic vascular resistance and microcirculation, for example, markers of endothelial state and inflammation, are perceived unchanged by 7 days of DI (Tomilovskaya et al., 2019; Amirova et al., 2020). The microbiome, as one of the important components of human metabolic homeostasis and immunity (Turroni et al., 2020), proved rather stable at the early stages of under analog microgravity experiments (Jin et al., 2019). Therefore, one cannot expect a substantial contribution of the gut-brain axis in subjects with PD specifically under short-term DI sessions.

Several similar to DI water-related rehabilitation approaches are currently used either for PD or arterial hypertension, for example, the so-called “passive heat therapy” which stands for warm water immersion (Brunt et al., 2016; Masiero et al., 2019; Sugawara and Tomoto, 2020), and “aquatic therapy” that is exercising in thermoneutral water (Carroll et al., 2017; Kim et al., 2018; Sato et al., 2019). Most of these studies reported the beneficial effect of water immersion on BP and HR. The study of Parker et al. (2018) reported on the modest increase of the variability of heart rate in healthy subjects under immersion in warm water (at 36°C), although this study has not specified the parameters of HRV.

Therefore, given that (1) either warm water immersion or aquatic therapy exerts beneficial effects on hemodynamics and HRV in healthy subjects and on hemodynamics in subjects with PD, (2) single one short-term DI session exerts an effect on blood pressure in PD, and (3) DI strongly affects hemodynamics and HRV in healthy subjects, we hypothesized that HRV and hemodynamics in subjects with PD would have also been modulated under conditions of DI. Additionally, as non-linear parameters are highly sensitive to the temporal structure of a signal, we also aimed at evaluating the effect of DI on autonomic regulation in PD with both linear and non-linear parameters of HRV.

## Materials and Methods

### Participants

The study enrolled 20 subjects with PD, 13 men and seven women. These subjects have participated in our previously published studies (Meigal et al., 2018, 2021a,b; Miroshnichenko et al., 2018), where one can find their individual clinical and anthropological characteristics and medication. General characteristic of the subjects is presented in Table 1. Individual data is presented in Supplementary Table 1. One man subject participated five times and another man subject participated three times within the years 2016–2019. A total of 26 DI courses were conducted.

**Table 1:** The anthropologic and clinical data of the subjects with PD.

	**Men (*n* = 13)**	**Women (*n* = 7)**
Age, years	58 ± 8	64 ± 4
Height, m	1.79 ± 0.06	1.58 ± 0.05
Weight, kg	77.1 ± 10.3	76.6 ± 10.1
Stage by Hoehn & Yahr	1–3	
Disease duration, years	3–6	
LED^*^, mg/day	344–763	

* *LED (levodopa equivalent dose) was calculated with the formula of Nutt et al. (2003)*.

The general inclusion criterion was the verified diagnosis of PD. The non-inclusion criteria for the DI group included a variety of pathologies that potentially could have worsened under DI, e.g., epilepsy, administration of muscle relaxants, hypovolemia, atrial fibrillation, hemorrhage of various etiology, lung diseases in the acute stage, myocardial infarction, oncologic problems, and blood clotting disorders such as phlebothrombosis or thrombophlebitis (Tomilovskaya et al., 2019). None of the subjects had brain trauma in anamnesis, including those associated with such sports as boxing and football. The study involved only patients who did not require drugs with a notable effect on autonomic regulation and/or cardiac function, for example, β-adrenoblockers. The non-inclusion criterion for HRV measurements was cardiac arrhythmias. After a comprehensive verbal explanation, all participants signed an informed consent form to participate. The study protocol was approved by the joint Ethics committee of the Ministry of Health care of the Republic of Karelia and Petrozavodsk State University (Statement of approval No. 31, 18.12.2014). Before the program of DI sessions, all PD subjects were examined with an active orthostatic test for orthostatic tolerance. In none of the subjects, orthostatic hypotension was found (Gerasimova-Meigal et al., 2021).

### The DI Intervention

The condition of DI was induced by means of the “Medical Facility of Artificial Weightlessness” (MEDSIM, Center for Aerospace Medicine and Technologies, State Scientific Center of Russian Federation “Institute of Biomedical Problems,” Moscow, Russia) housed in Petrozavodsk State University (Petrozavodsk, Russia). A detailed description of the procedure of DI applied to the group of PD subjects is available in our earlier papers (Meigal et al., 2018, 2021a,b; Miroshnichenko et al., 2018). The short-term DI session lasted 45-min. The DI session was carried out “on-medication,” starting at 9:30 AM. To synchronize the effects of anti-PD therapy with that of DI, subjects took their medicines 2 h before the study, at 7: 30 AM. During DI, subjects were lying in a head-out-of-water supine position in a bathtub with water individually adjusted for thermal comfort (32–34°C). One day prior to the study, the subjects underwent trial 15-min DI to identify hemodynamic changes during immersion and to get familiarized with the procedure. The program of DI comprised seven short-term DI sessions, conducted twice a week (total DI dose was 5.25 h), within 25–30 days (every 3–4 days).

### Outcome Measures

Within one DI session, data were collected before (baseline test), on the 15, 30, and 40 min of DI session. Across the program of DI, data were collected at the 1st, 4th, and 7th DI sessions.

Systolic and diastolic BP and HR were measured with UA-705 digital tonometer (A&D Company Ltd., Japan). ECG was recorded in the standard lead II for 5 min with the “VNS-Spectr” device (Neurosoft Ltd., Ivanovo, Russia). All ECG records were visually inspected for stationarity, and all artifacts were manually corrected. Only ECG records without arrhythmias on ECG (5-min long) were considered for HRV analysis. Further, HRV analysis was performed in accordance with international standards of measurement, physiological interpretation, and clinical use (Heart rate variability, 1996; Shaffer and Ginsberg, 2017). Linear (time- and frequency-domain) and non-linear (correlation dimension, entropies, DFA1 and DFA2, and percent of determinism and recurrence) were computed with Kubios Standard version 2 software (University of Eastern Finland, Kuopio, Finland; Tarvainen et al., 2014).

Time-domain HRV parameters included the HR, MeanRR, standard deviation (SDNN), root mean squared difference (RMSSD), the proportion of successive intervals >50 ms (pNN 50%) of normal RRi (NN), and triangular interpolation of the RR histogram index (TINN).

Frequency-domain HRV parameters included the total power (TP) spectrum of RRi, power spectrum at very low (VLF; <0.04 Hz), low (LF;0.04–0.15 Hz), and high-frequency bands (HF;0.15–0.40 Hz), the LF/HF ratio, and spectrum structure (% VLF, % LF, % HF, LF n.u, HF n.u.).

The analysis of non-linear HRV parameters included estimation of the indices of the Poincaré ellipse (SD1 and SD2) and recurrence plot (Mean line length—Lmean, Max line length—Lmax, recurrence rate—REC, determinism—DET, Shannon entropy—ShanEn), and others parameters approximated (ApEn) and sample (SampEn) entropy, parameters of detrended fluctuation analysis (DFA) with self-similarity indices for short (α1) and longtime intervals (α2), and correlation dimension (D2) (Voss et al., 2009; Tarvainen et al., 2014; Shaffer and Ginsberg, 2017).

### Statistical Analysis

Data were analyzed using IBM SPSS Statistics 21 software (SPSS, IBM Company, Chicago, IL, USA). Within one DI session, the SPSS Friedman test with further *post-hoc* comparisons (the Newman-Keuls test) was applied to find the differences between hemodynamic and HRV parameters at study points. Across the program of DI, the difference between studied parameters was estimated with ANOVA and non-parametric correlation (Spearmen test). The interaction between the parameters of hemodynamics and autonomic regulation within one DI session was assessed using the non-parametric correlation analysis (Spearmen test) and regression analysis. The results were considered significant at *p* < 0.05.

## Results

**Figure 1:**
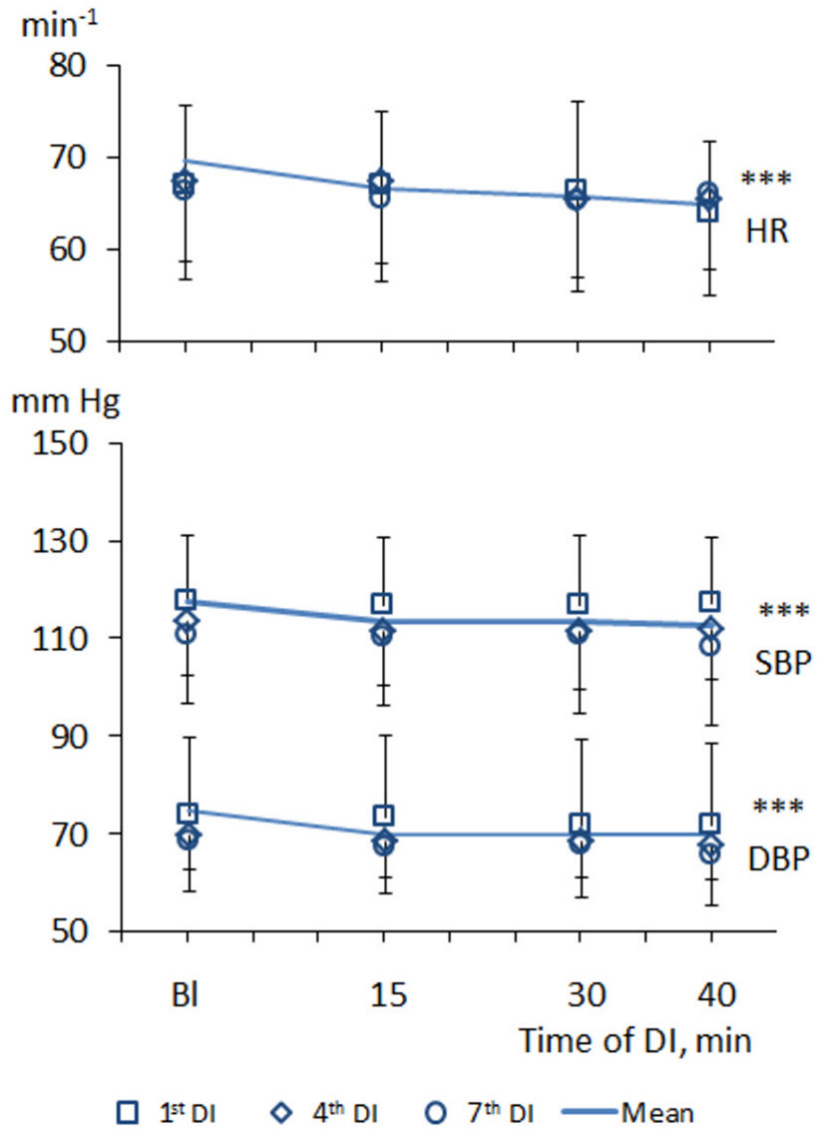
Dynamics of heart rate and blood pressure in PD patients during repeated “dry” immersion sessions. HR, heart rate; SBP, systolic blood pressure; DBP, diastolic blood pressure. The blue line represents the mean of tree studied DI sessions. The difference from the baseline condition ***p < 0.001 (Friedman test).

Within one DI session, the values of BP and HR tended to decrease (Figure 1). Thus, at baseline condition, systolic and diastolic BP was 117 ± 11 and 73 ± 7 mm Hg on average, respectively, while HR was 70 ± 9 min^−1^. Under one DI session, both systolic and diastolic BP has decreased by 5–7 mm Hg, and HR has decreased by 6–8 min^−1^ (*p* < 0.001). No difference between men and women subjects was found, which allowed forming one unified study group.

Across the program of DI sessions, the modest hypotensive effect was also the characteristic. Before the 1st DI, systolic BP was, on average, 118 ± 13 mm Hg, and diastolic BP – 72 ± 8 mm Hg. Before the 7th DI session, systolic BP was 111 ± 14 mm Hg (*p* < 0.001), and diastolic BP was 68 ± 8 mm Hg (*p* < 0.001).

Table 2 summarizes the results of HRV analysis within the DI session presented as Me (25%; 75%). At baseline condition, time-domain HRV parameters (SDNN, RMSSD, and pNN50) corresponded to low variability of HR, which, in turn, indicated reduced parasympathetic control of the heart rhythm (Heart rate variability, 1996; Shaffer and Ginsberg, 2017). The frequency-domain HRV parameters (TP and its LF and HF components) were decreased, which reflects the general deficit of the autonomic neurogenic control of HR. The ratio of main frequency domains was 56–27–17% (VLF > LF > HF), which indicated the predominance of humoral metabolic factors in the regulation of HR.

**Table 2:** Heart rate variability parameters in PD subjects under short-term DI sessions (average of all studied DI sessions).

**Parameter**	**Baseline**	**DI:15**	**DI:30**	**DI:40**	**Significance^a^**
**Time-domain HRV parameters**					
MeanRR, ms	885.4 (794.3; 960.1)	931.7 (851.1; 999.3)^***^	939.5 (885.9; 996.9)^***^	952.1 (893.2; 1011.3)^***^	<0.001
SDNN, ms	24.7 (19.0; 32.1)	28.9 (21.1; 41.2)^**^	35.3 (26.0; 48.0)^***^	37.4 (23.6; 45.9)^***^	<0.001
RMSSD, ms	16.6 (10.5; 20.0)	19.4 (13.2; 25.9)	23.5 (14.4; 34.4)	24.3 (15.0; 30.4)	
pNN50, %	0.6 (0.0; 1.8)	1.3 (0.0; 4.6)	2.9 (0.3; 8.0)^***^	3.2 (0.4; 7.4)^***^	<0.001
TINN, ms	110.0 (75.0; 145.0)	110.0 (77.5; 145.0)	122.5 (83.8; 181.3)^*^	115.0 (77.5; 162.5)	<0.05
**Frequency-domain HRV parameters**					
TP, ms^2^	406 (208; 705)	786 (330; 1596)^***^	1133 (479; 1959)^***^	1076 (418; 2133)^***^	<0.001
VLF, ms^2^	223 (129; 389)	324 (164; 849)	507 (249; 1013)^***^	594 (230; 890)^***^	<0.001
LF, ms^2^	100 (44; 211)	198 (80; 413)^***^	238 (106; 594)^***^	259 (109; 729)^***^	<0.001
HF, ms^2^	58 (30; 128)	118 (55; 244)^***^	156 (80; 259)^***^	189 (79; 275)^***^	<0.001
LF/HF	1.562 (1.001; 2.463)	1.659 (1.101; 2.874)	1.94 (0.98; 2.91)	1.837 (1.049; 3.223)	
VLF, %	57.3 (45.7; 70.3)	52.5 (35.5; 68.5)	53.5 (33.6; 68.2)	52.2 (40.4; 60.4) ^*^	<0.05
LF, %	23.2 (17.5; 30.5)	26.6 (19.0; 40.6)	27.0 (19.6; 39.4)	30.9 (19.8; 38.7)	
HF, %	15.0 (10.2; 20.3)	15.3 (9.1; 27.0)	14.2 (9.3; 26.8)	16.9 (11.1; 25.6)	
LF, n.u.	61.8 (50.3; 71.2)	62.4 (52.4; 74.2)	36.5 (25.6; 50.4)	64.8 (51.2; 76.3)	
HF, n.u.	38.2 (28.8; 49.7)	37.6 (25.8; 47.7)	53.5 (33.6; 68.2)	36.1 (23.7; 48.8)	
**Non-linear HRV parameters**
SD1, ms	11.9 (7.5; 14.4)	13.7 (9.4; 18.3)^***^	16.4 (9.8; 24.4)^***^	16.9 (10.6; 21.3)^***^	<0.001
SD2, ms	33.7 (23.8; 43.6)	37.0 (28.7; 54.6)	46.1 (33.7; 62.8)	51.1 (30.1; 63.4)	
Lmean, beats	14.15 (10.90; 17.71)	12.95 (9.60; 16.33)^*^	13.23 (10.77; 18.13)	11.90 (9.55; 15.45)^**^	<0.001
Lmax, beats	269 (190; 324)	206 (128; 286)^*^	222 (133; 296)	201 (128; 274)^***^	<0.001
REC, %	41.06 (35.32; 46.79)	39.99 (29.43; 45.18)	40.32 (32.35; 46.55)	37.00 (31.15; 43.27)	
DET, %	98.97 (98.13; 99.42)	98.69 (97.56; 99.43)	99.04 (98.42; 99.48)	98.74 (97.73; 99.34)	
ShanEn	3.409 (3.181; 3.645)	3.333 (3.032; 3.575)	3.336 (3.144; 3.607)	3.279 (3.024; 3.535)	
ApEn	1.119 (1.046; 1.160)	1.085 (1.017; 1.146)	1.095 (1.026; 1.137)	1.102 (1.039; 1.129)	
SampEn	1.482 (1.274; 1.700)	1.477 (1.225; 1.642)	1.421 (1.224; 1.590)	1.508 (1.298; 1.652)	
DFA:α1	1.162 (0.976; 1.313)	1.163 (0.944; 1.359)	1.185 (0.943; 1.357)	1.161 (1.051; 1.312)	
DFA:α2	1.007 (0.872; 1.100)	0.986 (0.765; 1.104)	0.977 (0.805; 1.097)	1.014 (0.863; 1.082)	
D2	0.341 (0.121; 0.700)	0.624 (0.237; 1.230)^***^	0.800 (0.480; 1.950)^***^	0.968 (0.361; 2.492)^***^	<0.001

a *The significance is based on Friedman test with further post-hoc comparisons (the Newman-Keuls test); the difference from the baseline condition:*

* *p < 0.05*,

** *p < 0.01*,

*** *p < 0.001*.

Within one DI session, the marked increase of both time- (SDNN, RMSSD, and pNN50) and frequency-domain (TP and its VLF, LF, and HF components) HRV parameters was found on the 15, 30, and 40th min of DI (see Table 2). This indicated the autonomic neurogenic parasympathetic and sympathetic response to DI. Nonetheless, the structure of the HRV spectrum did not significantly change except for a slight decrease in the VLF percentage. Similarly, no changes in entropy (ShanEn, ApEn, and SampEn) and DFA indices were found within one DI session. This evidenced that the time structure of the regulatory influence on the heart rate stood unchanged. The dynamics of HRV parameters were highly reproducible, i.e., it was almost identical among all sessions of DI.

**Figure 2:**
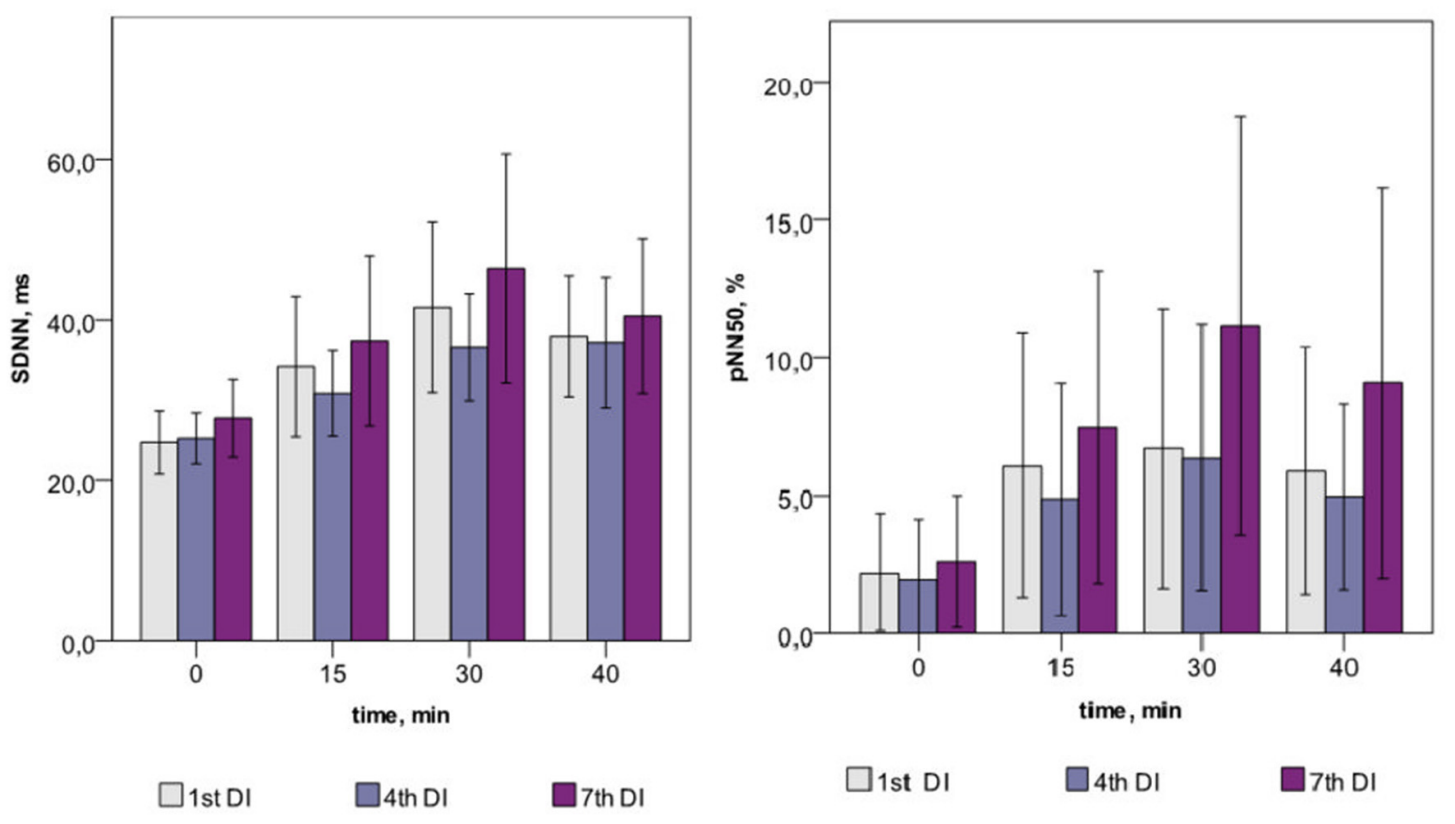
Time-domain HRV parameters in PD patients during repeated “dry” immersion sessions.

**Figure 3:**
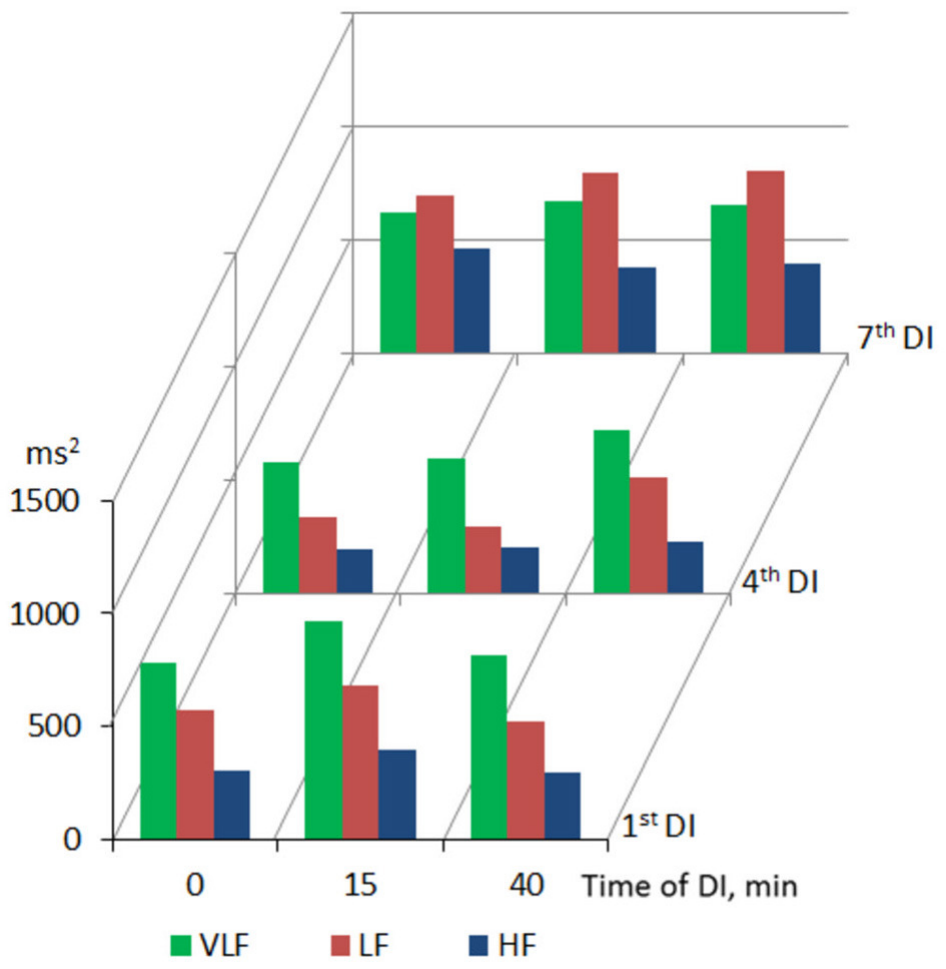
Frequency-domain HRV parameters in PD patients during repeated “”dry” immersion sessions.

**Figure 4:**
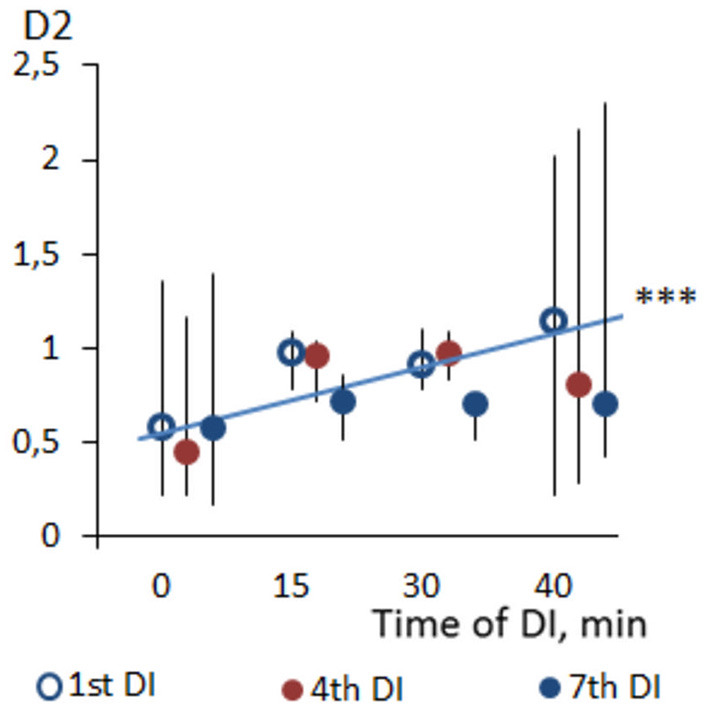
Dynamics of correlation dimension (D2) of HRV in PD patients during repeated “dry” immersion sessions. The blue line represents the regression line of D2 within the DI session (average of three studied DI sessions). ***p < 0.001 (Friedman test).

Across the program of DI sessions, no significant change of HRV parameters was found. The dynamics of time-domain HRV parameters (SDNN and pNN50) are presented in Figure 2, the HRV spectrum is shown in Figure 3, and D2 is shown in Figure 4. The main effect and interaction by short-term DI sessions and a course of DI on hemodynamics and HRV parameters (general linear model) are presented in Supplementary Table 2.

**Figure 5:**
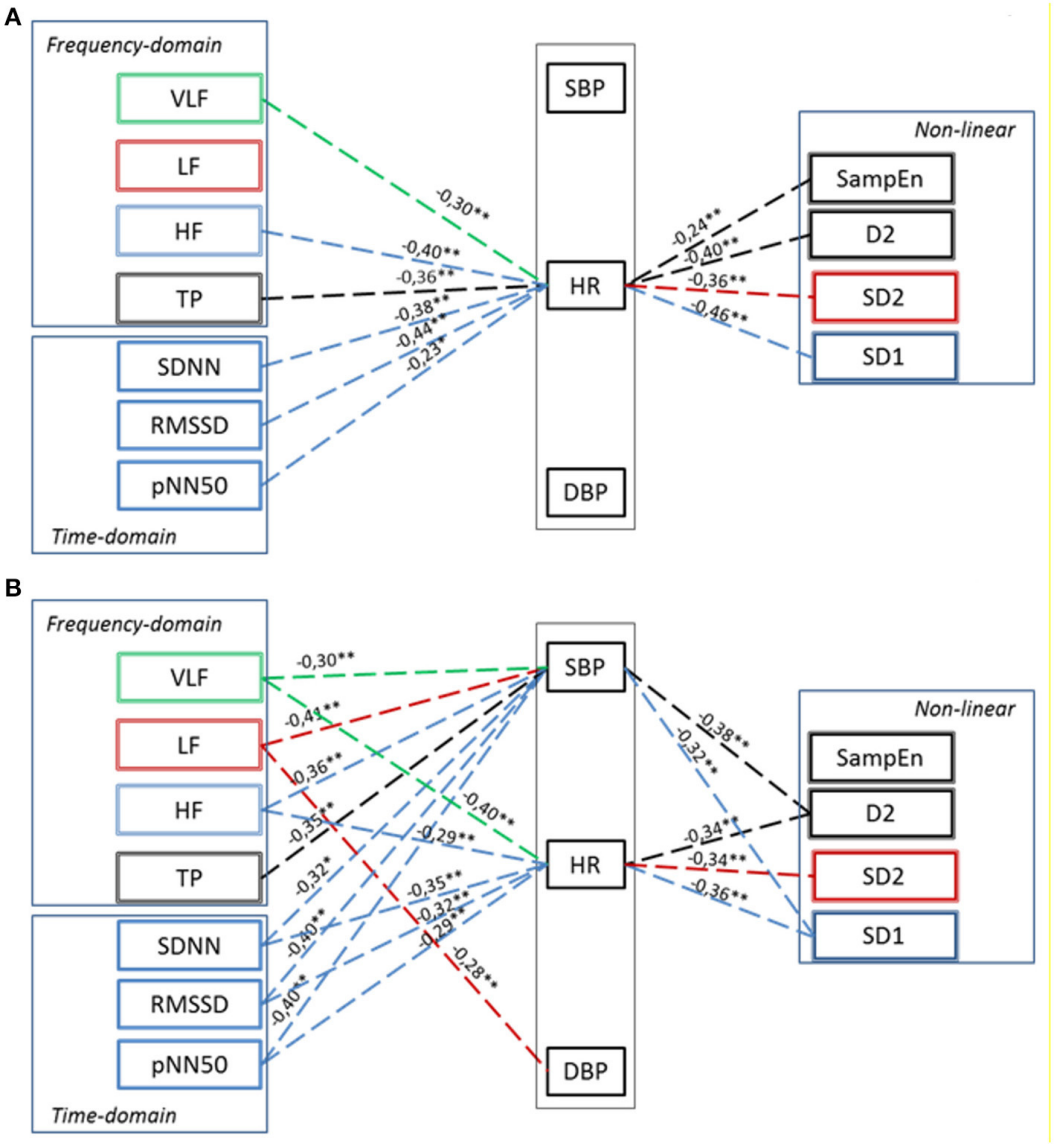
Correlation between Hemodynamics and HRV parameters in PD patients in baseline conditions **(A)** and at 40th minute of “dry” immersion session **(B)**. HR, heart rate; SBP, systolic blood pressure; DBP, diastolic blood pressure. Dashed line—the negative correlation. Significance of Spearman correlation coefficient: *p < 0.05, **p < 0.01.

The interaction between hemodynamics and autonomic regulation was assessed using correlation and regression analysis. At baseline condition, few correlations were found between HRV and HR, but not BP (Figure 5). Namely, there was a negative correlation between the HR and HRV indices of the parasympathetic activity. At the 40th min of DI, more correlations were found between the parameters of HRV and HR and BP (see Figure 5). The results of regression analysis were in line with that of correlation analysis, indicating the same associations between parameters (Supplementary Table 3). This indicated the emergence of systemic compensatory mechanisms which help to maintain BP in PD subjects under DI.

## Discussion

According to the working hypothesis of this study, in subjects with PD under conditions of DI one could expect the decrease of BP and modifications of autonomic regulation seen with HRV parameters. In line with this hypothesis, we found that both systolic and diastolic BP, indeed decreased within one DI session, by some 5–7 mm Hg, and HR, by 6–8 min^−1^. In the program of DI sessions, both diastolic and systolic BP has significantly decreased by 8–10 mm Hg on the fourth DI session. Most of HRV time-domain parameters (SDNN and pNN50) and the power of all bands of the frequency spectrum (TP, VLF, LF, and HF) have significantly increased within a single DI. Also, there was a notable tendency of VLF to decrease by the 40th min of a single DI session. As for the non-linear parameters of HRV, only correlation dimension (D2) and SD1 have significantly increased along with the DI session. By contrast, all kinds of entropy and DFA indices, and recurrent rates did not respond to the conditions of DI. Across the DI program, none of the HRV parameters has been significantly modified. Altogether, these data suggested that in subjects with PD the autonomic cardiac regulation and hemodynamics are strongly and beneficially modified within a 45-min DI session, but these parameters remained largely unchanged across the program of seven DI sessions. Several factors may have contributed to such outcome, among which are (1) ambient temperature, (2) conditions of immersion *per se*, (3) baroreceptor reflex sensitivity (BRS), and (4) PD-specific impairment of the autonomic nervous system.

### Hemodynamics

#### The “Warm Water” Immersion Effect

The conditions of DI comprised of two major physical factors, namely, immersion and temperature. These factors probably differentially contributed to the result. Some studies report that even a short-term immersion in water with a temperature up to 40°C can provoke the so-called “warm water” effect in a form of mental and muscular relaxation in PD (Masiero et al., 2019). Even 5-min warm water immersion significantly improved arterial wall stiffness what lasts for at least 15 min after the session of warm water immersion (Sugawara and Tomoto, 2020). In the study of Brunt et al. (2016), the program of five 1-h warm water immersion at 40.5°C within 8 weeks (“passive heat therapy”) significantly increased flow-mediated arterial dilatation, reduced arterial stiffness mean arterial and diastolic BP, and carotid intima-media thickness in young healthy subjects. In the present study, the water temperature at DI was 32°C, which can generally be considered as thermoneutral. Therefore, it is not likely that temperature was the leading factor in BP modification. However, the separate effect of DI, “no-dry” water immersion, and water temperature is still to be evaluated (Sugawara and Tomoto, 2020).

#### Water Immersion Effect

Conditions, which are similar to the program of DI or sessions of warm water immersion, are represented by varied protocols of so-called “aquatic therapy,” which appear as physical exercising in a water pool at thermoneutral water temperature. In most studies with these protocols, both systolic and diastolic BP has decreased by 10 mm Hg just some minutes after immersion in water (Ward et al., 2005; Júnior et al., 2020). Aquatic therapy is widely used to treat mental and motor disorders in PD (Carroll et al., 2017; Kim et al., 2018; Sato et al., 2019). Surprisingly, hemodynamics and HRV in subjects with PD under aquatic therapy appear largely uninvestigated.

#### Dry Immersion Effect

There is a lack of studies on cardiovascular responses in humans to a very short (within 1 h) DI session. Still, it is known that in healthy subjects, during the 1st h of DI, total peripheral resistance has decreased by 7%, which led to diastolic BP decreased by 5 mm Hg, and HR by 5 min^−1^ (Ogoh et al., 2017; Navasiolava et al., 2020). Similar results were reported in the study by Meigal et al. (2020). We regarded that decrease of the total peripheral vascular resistance has contributed to the decrease of BP in subjects with PD. Altogether, we regarded that the effect of DI on subjects with PD can be attributed to the condition of immersion to water.

### HRV Parameters

#### Baroreceptor Reflex Sensitivity

Baroreceptor-heart rate reflex sensitivity (BRS) appears as an important determinant of the short-term regulation of BP (Nasr et al., 2005). In healthy subjects, conditions of DI provoked attenuation of BRS what was seen as increased orthostatic hypotension and, hence, decreased orthostatic tolerance (Tomilovskaya et al., 2019; Borovik et al., 2020). However, this result was obtained after much longer DI sessions (3–21 days). In subjects with PD, BRS is substantially decreased, which is associated with orthostatic hypotension (Blaho et al., 2017; Gerasimova-Meigal et al., 2021), arterial stiffness, presence of central α-synuclein aggregation, cardiac sympathetic denervation, attenuated muscle sympathetic nerve activity (Sabino-Carvalho et al., 2021). In our study, none of the subjects with PD had signs of orthostatic hypotension what was earlier reported in the study by Gerasimova-Meigal et al. (2021). Nonetheless, attenuation of BRS may still have contributed to the decrease of BP in subjects with PD under DI sessions.

#### Baseline HRV Data in PD

Before the program of DI sessions, HRV was clearly reduced in subjects with PD, according to decreased values of time- and frequency-domain parameters. This indicated the decrease of *both* sympathetic and parasympathetic autonomic activity, which is consistent with earlier numerous studies (Jain, 2011; Jain and Goldstein, 2012; Soares et al., 2013; Maetzler et al., 2015; Gibbons et al., 2017; Akbilgic et al., 2020; Li et al., 2020). The low value of the parasympathetic-linked HRV parameters might evidence attenuated cardiorespiratory coupling (Heart rate variability, 1996; Shaffer and Ginsberg, 2017). As for non-linear parameters of HRV, indices, which characterized parasympathetic and sympathetic nervous activity (SD1 and SD2, correspondingly), were reduced in PD in comparison with older non-PD subjects (Kallio et al., 2002; Voss et al., 2009). Correlation dimension (D2), which characterized self-similarity of a signal, or the number of regulation inputs (differential equations) was markedly decreased in PD subjects in comparison with that of non-PD subjects (Acharya et al., 2014). Surprisingly, entropy and DFA indices, and recurrence rate of PD subjects generally fitted values regarded as normal for healthy older people (Voss et al., 2009; Acharya et al., 2014). Altogether, HRV signal in PD subjects can be regarded as less complex in comparison to age-matched healthy controls, which accords with evidence of decreased autonomic control at PD, presented by traditional HRV parameters.

#### HRV During a Single One DI Session

Within one DI session, almost all time- and frequency-domain HRV parameters have significantly increased. For example, the value of pNN50 has increased by three times by the end of the DI session. Such a result represents the growing variability of HR under the conditions of DI in PD subjects. As for non-linear parameters, SD1, which informs on the parasympathetic control of the heart, has significantly increased from 7–14 to 10–21, which is close to the values of healthy older subjects (Voss et al., 2009). The correlation dimension of HRV has also increased by three times, which indicates on growing complexity of the signal and, hence, emerging regulating inputs. We assume that such inputs could be associated with BRS and, emerging cardiorespiratory coupling, therefore, increased parasympathetic and sympathetic nervous control of HRV. A similar phenomenon of stronger cardiorespiratory coupling we reported in our earlier study with a deep breathing test (Gerasimova-Meigal et al., 2021). Altogether, the HRV of PD subjects after a 45-min DI session has clearly shifted in the direction of “normal” age-matched values. Such modification was possibly provoked by the “centralization” of circulation due to the compression effect of DI on peripheral tissue (Navasiolava et al., 2011; Tomilovskaya et al., 2019; Pandiarajan and Hargens, 2020).

#### HRV During the Program of DI Sessions

Compared with one DI session (acute effect), the effect of the program of DI sessions on HRV parameters was strikingly weak. This evidences the limited capability of the autonomic nervous system in PD to restore its functioning, presumably due to profound neurodegeneration. In turn, it indicates reduced mechanisms of neuroplasticity in PD. Similarly, in our earlier study, we have demonstrated no HRV dynamics seen with cardiovascular tests during the program of DI (Gerasimova-Meigal et al., 2021). Such results accord with the study of Rocha et al. (2018) in which a program of game-based rehabilitation in PD presented no modification of HRV. Traces of adaptation are usually seen after repetitive exposures (pre-conditioning), which are usually associated with intensive metabolic response to the intervention, e.g., cold, hypoxia, or physical exercise. Unlike pre-conditioning, DI appears as a rather deconditioning factor (Acket et al., 2018). We presume that the absence of long-term modification of the studied parameters can be attributed to the different environmental effects of DI. Additionally, limited modification of the studied parameters to the program of DI sessions could be linked to the limited adaptation capacity of the organism of a PD subject.

#### Correlation Between BP, HR, and HRV

The phenomenon of cardiovascular coupling is well-known and documented. For example, both feedback (from BP on HR, *via* the mechanism of BRS), and feedforward (from HR on BP) influences are known to take place (Schulz et al., 2013). However, in patients with PD, we did not find a correlation between BP and HR at baseline conditions, which might inform on cardiovascular uncoupling in PD. This could well-originate from reduced BRS, which is the characteristic of PD. Still, there was a correlation between HR and almost all HRV parameters. That is not surprising because HR appeared as a kind of “parent” signal for all HRV parameters. Also, the correlation between HR and HRV indices of parasympathetic activity (SDNN, RMSSD, pNN50, TP, HF, and SD1) was negative, which is consistent with consensus on autonomic control of the cardiovascular system (Heart rate variability, 1996; Shaffer and Ginsberg, 2017). The negative correlation between hemodynamic parameters and HRV indices of sympathetic activity (LF and SD2) did not fit this consensus, probably due to impaired sympathetic control and baroreflex sensitivity, which is often observed in PD patients (Blaho et al., 2017; Gerasimova-Meigal et al., 2021).

By contrast, under the conditions of DI significant correlations appeared between HRV parameters and systolic BP, which evidenced the temporal emergence of causal coupling between BP and HR. That is in line with the data on modification of values of HRV parameters during the DI session. In a way, during DI the cardiovascular system of PD subjects looks more susceptive to regulation.

## Conclusion

At baseline condition, time- and frequency-domain HRV parameters in subjects with PD showed low variability of HR, which indicates its reduced autonomic neurogenic control. The temporal structure of the regulatory input to the heart seen with non-linear parameters of HRV was characterized by low complexity. Within one DI session, systolic and diastolic BP has modestly decreased, and time- and frequency-domain parameters of HRV have significantly increased, what altogether evidenced by compensatory hemodynamics mechanisms during DI. Across the program of DI sessions, the hypotensive effect was also present, but no notable modification of the HRV-parameters was found. The absence of long-term modification of the studied parameters can be attributed both to deconditioning effect of the DI, and limited adaptation to DI in subjects with PD due to neurodegeneration. That should be taken into consideration when planning either rehabilitation measures in subjects of older age with modeled microgravity or space flights in older candidates.

## Limitations

There were several limitations to our study. First, an adequate age-matched control group would hardly be formed, because all potential older subjects are characterized by multimorbidity. Therefore, our study was designed only as a self-control one. Second, we experienced some difficulties with recruiting subjects to the study, as many of the candidate subjects did not meet strict inclusion criteria due to health status, especially cardiac arrhythmias, which did not allow using HRV analysis for assessment of autonomic regulation.

## Prospective

For a better insight into the mechanisms of the autonomic nervous regulation in PD subjects under DI, one should consider investigating (1) cardiorespiratory coupling of HRV during DI session and (2) to evaluate HRV, BP, and HR in healthy controls during a short-term DI session to make comparison with the group of PD subjects. In addition to the main purpose of this study, we are convinced that it can also contribute to the field of space physiology since all the subjects in our study were elderly people who are expected to take on a significant proportion of commercial space travel in the future. Space tourism will evolve toward suborbital space flights, which are expected to last for shorter periods of time. For example, Amazon CEO Jeff Besos recently announced that an 82-year-old woman will join him on a space trip (Wattles, 2021b), and Richard Bronson has already performed such a trip (Wattles, 2021a). Thus, this study closed an important gap in ground-based space experiments, since most of them last for longer periods (several hours, days, weeks) and attract mostly younger healthy subjects. For this reason, this study is one of the few exploratory ones in a row with our previous studies (Meigal et al., 2018, 2020, 2021a,b) in space neuroscience and space suborbital tourism.

## Data Availability Statement

The raw data supporting the conclusions of this article will be made available by the authors, without undue reservation.

## Ethics Statement

The studies involving human participants were reviewed and study protocol was approved by joint Ethic Committee of the Ministry of Health care of the Republic of Karelia and Petrozavodsk State University (Statement of approval No. 31, 18.12.2014). The patients/participants provided their written informed consent to participate in this study.

## Author Contributions

LG-M contributed to the basic concept of the study, study design and implementation (supervising the DI procedure, monitoring the ECG, blood pressure, and clinical condition of the subjects during DI), the statistical analysis, the interpretation of results, writing the manuscript, and approval of the final draft. AM contributed to the basic concept of the study, study design, statistical analysis, interpretation of results, writing the manuscript, and approval of the final draft. NS contributed to the study design and implementation (supervising the DI procedure, monitoring the ECG, blood pressure of the subjects during DI, and data analyses), the interpretation of results, and approval of the final draft. IS contributed to the basic concept of the study, study design, the interpretation of results, and approval of the final draft. All authors contributed to the article and approved the submitted version.

## Funding

This research was financially supported by the Ministry of Science and Higher Education of the Russian Federation (Theme No. 0752-2020-0007).

## Conflict of Interest

The authors declare that the research was conducted in the absence of any commercial or financial relationships that could be construed as a potential conflict of interest.

## Publisher's Note

All claims expressed in this article are solely those of the authors and do not necessarily represent those of their affiliated organizations, or those of the publisher, the editors and the reviewers. Any product that may be evaluated in this article, or claim that may be made by its manufacturer, is not guaranteed or endorsed by the publisher.
