# Supplementary material for: Autonomic Function in Parkinson's Disease Subjects Across Repeated Short-Term Dry Immersion: Evidence From Linear and Non-linear HRV Parameters
Source: Front Physiol. 2021 Oct 6;12:712365. doi: 10.3389/fphys.2021.712365 (PMC8526731; doi:10.3389/fphys.2021.712365)
Supplement: Supplementary file 1 [file Data_Sheet_1.docx]

Table S1. The anthropologic and clinical data on the subjects with PD.

| **No. of subject** | **Age (yr) and gender** | **Height (cm), weight (kg)** | **Disease duration (yr)** | **Stage by H & Y** | **Clinical**  **form** | **LED * (mg/day)** |
| --- | --- | --- | --- | --- | --- | --- |
| 1 | 70 M | 176, 74 | 2 | 3 | PD, T | 187,5 |
| 2 | 68 M | 167, 78 | 3 | 2 | PD, AR | 375 |
| 3 | 47 M | 182, 81 | 3 | 1 | PD, AR | No medication |
| 4 | 58 M | 170, 60 | N/A | 3 | PD, T | 675 |
| 5 | 69 M | 179, 69 | 4 | 3 | PD, T | 250 |
| 6 | 65 F | 152, 78 | 7 | 1 | VP, AR | 1100 |
| 7 | 61 M | 188, 83 | 6 | 1 | PD, T | 312,5 |
| 8 | 58 F | 158, 65 | N/A | 2 | PD, T | No levodopa based treatment |
| 9 | 50 М | 171, 94 | 4 | 2 | PD, T | 450 |
| 10 | 55 М | 178, 86 | 6 | 2 | PD, T | 450 |
| 11 | 62 М | 180, 69 | 7 | 2 | PD, T | 869 |
| 12 | 64 F | 160, 81 | 4 | 2 | PD, T | 250 |
| 13 | 72 F | 154, 66 | 8 | 2 | VP, T | No levodopa based treatment |
| 14 | 71 М | 170, 51 | 8 | 2 | PD, T | No levodopa based treatment |
| 15 | 68 F | 164, 90 | 3 | 2.5 | VP, T | 178 |
| 16 | 73 M | 172, 61 | 8 | 3 | PD, T | 362 |
| 17 | 57 M | 178, 63 | 6 | 2.5 | PD, T | 406 |
| 18 | 66 F | 160, 67 | 2 | 2 | VP, T | No levodopa based treatment |
| 19 | 62 F | 165, 57 | 10 | 3 | PD, AR | 188 |
| 20 | 53 M | 171, 80 | 5 | 1.5 | PD, AR | 200 |

* LED (levodopa equivalent dose) was calculated with formula of Nutt et al. (2003). T - tremulous form, AR - akinetic-rigid form of PD, VP - vascular parkinsonism. N/A – not assessed.

Subject #3 participated 5 times within the years 2016-2019, subject #7 – 3 times within the years 2017-2019.

Subjects #11, 13, and 15 had diabetes mellitus type 2. These subjects were characterized high compliance to treatment and monitored there blood glucose concentration.

Table S2. The main effect and interaction by short-term DI session and a course of DI on hemodynamics and HRV parameters in PD subjects

| **Parameter** | **Factor** | **Sum of Squares** | **Degrees of Freedom** | **Mean Square** | **F** | ***p*** |
| --- | --- | --- | --- | --- | --- | --- |
| SBP, mm Hg | DI time | 1121,732 | 3 | 373,911 | 2,238 | 0,084 |
|  | DI course | 1911,456 | 2 | 955,728 | 5,720 | 0,004 |
|  | DI time x course | 222,498 | 6 | 37,083 | 0,222 | 0,969 |
|  | Error | 44108,591 | 264 | 167,078 |  |  |
| DBP, mm Hg | DI time | 1099,380 | 3 | 366,460 | 7,541 | 0,000 |
|  | DI course | 586,573 | 2 | 293,286 | 6,035 | 0,003 |
|  | DI time x course | 58,009 | 6 | 9,668 | 0,199 | 0,977 |
|  | Error | 12829,672 | 264 | 48,597 |  |  |
| HR, min^-1^ | DI time | 962,968 | 3 | 320,989 | 4,099 | 0,007 |
|  | DI course | 36,356 | 2 | 18,178 | 0,232 | 0,793 |
|  | DI time x course | 126,196 | 6 | 21,033 | 0,269 | 0,951 |
|  | Error | 19735,638 | 252 | 78,316 |  |  |
| **Time-domain HRV parameters** | | | | | | |
| MeanRR, ms | DI time | 179599,374 | 3 | 59866,458 | 4,952 | 0,002 |
|  | DI course | 10502,346 | 2 | 5251,173 | 0,434 | 0,648 |
|  | DI time x course | 16608,923 | 6 | 2768,154 | 0,229 | 0,967 |
|  | Error | 3046735,686 | 252 | 12090,221 |  |  |
| SDNN, ms | DI time | 9358,720 | 3 | 3119,573 | 8,556 | 0,000 |
|  | DI course | 1304,380 | 2 | 652,190 | 1,789 | 0,169 |
|  | DI time x course | 394,268 | 6 | 65,711 | 0,180 | 0,982 |
|  | Error | 91883,325 | 252 | 364,616 |  |  |
| RMSSD, ms | DI time | 5975,868 | 3 | 1991,956 | 6,024 | 0,001 |
|  | DI course | 1522,687 | 2 | 761,343 | 2,302 | 0,102 |
|  | DI time x course | 615,730 | 6 | 102,622 | 0,310 | 0,931 |
|  | Error | 83334,514 | 252 | 330,693 |  |  |
| pNN50, % | DI time | 1284,956 | 3 | 428,319 | 3,638 | 0,013 |
|  | DI course | 423,523 | 2 | 211,761 | 1,799 | 0,168 |
|  | DI time x course | 151,374 | 6 | 25,229 | 0,214 | 0,972 |
|  | Error | 29669,302 | 252 | 117,735 |  |  |
| TINN, ms | DI time | 38176,734 | 3 | 12725,578 | 2,196 | ,089 |
|  | DI course | 283,009 | 2 | 141,505 | ,024 | ,976 |
|  | DI time x course | 13232,445 | 6 | 2205,407 | ,381 | ,891 |
|  | Error | 1460016,128 | 252 | 5793,715 |  |  |
| **Frequency-domain HRV parameters** | | | | | | |
| TP, ms ^2^ | DI time | 82979040,159 | 3 | 27659680,053 | 4,404 | 0,005 |
|  | DI course | 19703640,218 | 2 | 9851820,109 | 1,568 | 0,210 |
|  | DI time x course | 14313274,946 | 6 | 2385545,824 | 0,380 | 0,892 |
|  | Error | 1582823818,555 | 252 | 6281046,899 |  |  |
| VLF, ms ^2^ | DI time | 8856603,888 | 3 | 2952201,296 | 6,422 | 0,000 |
|  | DI course | 66428,904 | 2 | 33214,452 | 0,072 | 0,930 |
|  | DI time x course | 252686,971 | 6 | 42114,495 | 0,092 | 0,997 |
|  | Error | 115837366,538 | 252 | 459672,089 |  |  |
| LF, ms ^2^ | DI time | 13950098,395 | 3 | 4650032,798 | 2,812 | 0,040 |
|  | DI course | 5723890,325 | 2 | 2861945,162 | 1,731 | 0,179 |
|  | DI time x course | 3210045,585 | 6 | 535007,598 | 0,324 | 0,924 |
|  | Error | 416672651,088 | 252 | 1653462,901 |  |  |
| HF, ms ^2^ | DI time | 5850972,669 | 3 | 1950324,223 | 2,603 | 0,052 |
|  | DI course | 3232370,068 | 2 | 1616185,034 | 2,157 | 0,118 |
|  | DI time x course | 3193339,507 | 6 | 532223,251 | 0,710 | 0,641 |
|  | Error | 188787871,478 | 252 | 749158,220 |  |  |
| LF/HF | DI time | 3,096 | 3 | 1,032 | 0,258 | 0,855 |
|  | DI course | 15,651 | 2 | 7,825 | 1,957 | 0,143 |
|  | DI time x course | 13,080 | 6 | 2,180 | 0,545 | 0,773 |
|  | Error | 1007,617 | 252 | 3,998 |  |  |
| VLF, % | DI time | 1797,834 | 3 | 599,278 | 1,552 | 0,201 |
|  | DI course | 87,803 | 2 | 43,902 | 0,114 | 0,893 |
|  | DI time x course | 780,672 | 6 | 130,112 | 0,337 | 0,917 |
|  | Error | 97276,106 | 252 | 386,016 |  |  |
| LF, % | DI time | 978,753 | 3 | 326,251 | 0,883 | 0,450 |
|  | DI course | 59,517 | 2 | 29,758 | 0,081 | 0,923 |
|  | DI time x course | 861,685 | 6 | 143,614 | 0,389 | 0,886 |
|  | Error | 93100,569 | 252 | 369,447 |  |  |
| HF, % | DI time | 439,972 | 3 | 146,657 | 0,821 | 0,483 |
|  | DI course | 3,175 | 2 | 1,588 | 0,009 | 0,991 |
|  | DI time x course | 749,994 | 6 | 124,999 | 0,699 | 0,650 |
|  | Error | 45032,179 | 252 | 178,699 |  |  |
| LF, n.u. | DI time | 228,827 | 3 | 76,276 | 0,236 | 0,871 |
|  | DI course | 434,552 | 2 | 217,276 | 0,671 | 0,512 |
|  | DI time x course | 625,388 | 6 | 104,231 | 0,322 | 0,925 |
|  | Error | 81564,942 | 252 | 323,670 |  |  |
| HF, n.u. | DI time | 205,294 | 3 | 68,431 | 0,210 | 0,890 |
|  | DI course | 395,206 | 2 | 197,603 | 0,606 | 0,546 |
|  | DI time x course | 699,059 | 6 | 116,510 | 0,357 | 0,905 |
|  | Error | 82167,678 | 252 | 326,062 |  |  |
| **Non-linear HRV parameters** | | | | | | |
| SD1, ms | DI time | 2620,534 | 3 | 873,511 | 5,309 | 0,001 |
|  | DI course | 564,919 | 2 | 282,459 | 1,717 | 0,182 |
|  | DI time x course | 368,964 | 6 | 61,494 | 0,374 | 0,895 |
|  | Error | 41462,134 | 252 | 164,532 |  |  |
| SD2, ms | DI time | 10238,181 | 3 | 3412,727 | 3,961 | 0,009 |
|  | DI course | 963,770 | 2 | 481,885 | 0,559 | 0,572 |
|  | DI time x course | 3405,105 | 6 | 567,518 | 0,659 | 0,683 |
|  | Error | 217103,671 | 252 | 861,523 |  |  |
| Lmean, beats | DI time | 223,336 | 3 | 74,445 | 1,714 | 0,165 |
|  | DI course | 14,043 | 2 | 7,021 | 0,162 | 0,851 |
|  | DI time x course | 68,321 | 6 | 11,387 | 0,262 | 0,954 |
|  | Error | 10944,170 | 252 | 43,429 |  |  |
| Lmax, beats | DI time | 87646,344 | 3 | 29215,448 | 3,047 | 0,029 |
|  | DI course | 1809,415 | 2 | 904,708 | 0,094 | 0,910 |
|  | DI time x course | 23234,242 | 6 | 3872,374 | 0,404 | 0,876 |
|  | Error | 2416461,002 | 252 | 9589,131 |  |  |
| REC, % | DI time | 751,958 | 3 | 250,653 | 1,981 | 0,117 |
|  | DI course | 56,117 | 2 | 28,058 | 0,222 | 0,801 |
|  | DI time x course | 219,382 | 6 | 36,564 | 0,289 | 0,942 |
|  | Error | 31889,215 | 252 | 126,545 |  |  |
| DET, % | DI time | 488,700 | 3 | 162,900 | 0,734 | 0,532 |
|  | DI course | 355,621 | 2 | 177,810 | 0,802 | 0,450 |
|  | DI time x course | 1183,528 | 6 | 197,255 | 0,889 | 0,503 |
|  | Error | 55897,095 | 252 | 221,814 |  |  |
| ShanEn | DI time | 0,790 | 3 | 0,263 | 1,801 | 0,147 |
|  | DI course | 0,045 | 2 | 0,023 | 0,155 | 0,856 |
|  | DI time x course | 0,427 | 6 | 0,071 | 0,486 | 0,819 |
|  | Error | 36,858 | 252 | 0,146 |  |  |
| ApEn | DI time | 1,407 | 3 | 0,469 | 1,090 | 0,354 |
|  | DI course | 0,743 | 2 | 0,372 | 0,864 | 0,423 |
|  | DI time x course | 2,121 | 6 | 0,353 | 0,822 | 0,554 |
|  | Error | 108,402 | 252 | 0,430 |  |  |
| SampEn | DI time | 0,272 | 3 | 0,091 | 0,944 | 0,420 |
|  | DI course | 0,072 | 2 | 0,036 | 0,373 | 0,689 |
|  | DI time x course | 0,044 | 6 | 0,007 | 0,076 | 0,998 |
|  | Error | 24,189 | 252 | 0,096 |  |  |
| DFA:α1 | DI time | 0,029 | 3 | 0,010 | 0,131 | 0,942 |
|  | DI course | 0,053 | 2 | 0,026 | 0,350 | 0,705 |
|  | DI time x course | 0,107 | 6 | 0,018 | 0,237 | 0,964 |
|  | Error | 18,964 | 252 | 0,075 |  |  |
| DFA:α2 | DI time | 0,097 | 3 | 0,032 | 0,744 | 0,527 |
|  | DI course | 0,052 | 2 | 0,026 | 0,601 | 0,549 |
|  | DI time x course | 0,093 | 6 | 0,016 | 0,359 | 0,904 |
|  | Error | 10,926 | 252 | 0,043 |  |  |
| D2 | DI time | 23,945 | 3 | 7,982 | 6,060 | 0,001 |
|  | DI course | 4,676 | 2 | 2,338 | 1,775 | 0,172 |
|  | DI time x course | 0,221 | 6 | 0,037 | 0,028 | 1,000 |
|  | Error | 331,937 | 252 | 1,317 |  |  |

Table S3. Coefficients of the linear regression model for the interaction between hemodynamic parameters and HRV at baseline and at the 40th minute of a short-term DI session in PD patients.

| **Dependent variable** | **Independent variable** | **Model** | **Unstandardized Coefficients** | | **Standardized Coefficient** | **t** | **Significance** |
| --- | --- | --- | --- | --- | --- | --- | --- |
|  |  |  | **B** | **Std. Error** | **Beta** |  |  |
| Baseline | | | | | | | |
| SBP, mm Hg | SDNN, ms | Constant | 122,057 | 3,824 |  | 31,916 | 0,000 |
|  |  | Slope | -0,191 | 0,139 | -0,164 | -1,380 | 0,172 |
|  | RMSSD, ms | Constant | 119,623 | 2,927 |  | 40,873 | 0,000 |
|  |  | Slope | -0,149 | 0,154 | -0,115 | -0,966 | 0,338 |
|  | pNN50, % | Constant | 117,220 | 1,471 |  | 79,665 | 0,000 |
|  |  | Slope | -0,047 | 0,256 | -0,022 | -0,185 | 0,854 |
|  | TP, ms^2^ | Constant | 117,713 | 1,916 |  | 61,442 | 0,000 |
|  |  | Slope | -0,001 | 0,002 | -0,053 | -0,442 | 0,660 |
|  | VLF, ms^2^ | Constant | 117,797 | 2,081 |  | 56,605 | ,000 |
|  |  | Slope | -0,002 | 0,005 | -0,052 | -0,432 | 0,667 |
|  | LF, ms^2^ | Constant | 117,637 | 1,618 |  | 72,712 | 0,000 |
|  |  | Slope | -0,003 | 0,005 | -0,071 | -0,588 | 0,559 |
|  | HF, ms^2^ | Constant | 118,337 | 1,528 |  | 77,460 | 0,000 |
|  |  | Slope | -0,010 | 0,006 | -0,191 | -1,620 | 0,110 |
|  | SD1 | Constant | 119,098 | 2,953 |  | 40,334 | 0,000 |
|  |  | Slope | -0,164 | 0,217 | -0,091 | -0,756 | 0,452 |
|  | SD2 | Constant | 116,548 | 2,041 |  | 57,098 | 0,000 |
|  |  | Slope | 0,015 | 0,040 | 0,044 | 0,369 | 0,713 |
|  | SampEn | Constant | 115,500 | 7,072 |  | 16,332 | 0,000 |
|  |  | Slope | 1,094 | 4,708 | 0,028 | 0,232 | 0,817 |
|  | D2 | Constant | 117,874 | 1,652 |  | 71,333 | 0,000 |
|  |  | Slope | -1,194 | 1,502 | -0,095 | -0,795 | 0,429 |
| DBP, mm Hg | SDNN, ms | Constant | 76,192 | 2,161 |  | 35,266 | 0,000 |
|  |  | Slope | -0,135 | 0,078 | -,204 | -1,729 | 0,088 |
|  | RMSSD, ms | Constant | 73,082 | 1,676 |  | 43,594 | 0,000 |
|  |  | Slope | -0,023 | 0,088 | -0,032 | -0,263 | 0,793 |
|  | pNN50, % | Constant | 72,448 | 0,835 |  | 86,811 | 0,000 |
|  |  | Slope | 0,107 | 0,145 | 0,088 | 0,737 | 0,464 |
|  | TP, ms^2^ | Constant | 72,740 | 1,092 |  | 66,605 | 0,000 |
|  |  | Slope | -8,503E-005 | 0,001 | -0,008 | -0,064 | 0,949 |
|  | VLF, ms^2^ | Constant | 73,482 | 1,180 |  | 62,294 | 0,000 |
|  |  | Slope | -0,003 | 0,003 | -0,106 | -0,883 | 0,380 |
|  | LF, ms^2^ | Constant | 72,499 | 0,922 |  | 78,604 | 0,000 |
|  |  | Slope | 0,001 | 0,003 | 0,045 | 0,376 | 0,708 |
|  | HF, ms^2^ | Constant | 72,851 | 0,885 |  | 82,300 | 0,000 |
|  |  | Slope | -0,001 | 0,004 | -0,044 | -0,366 | 0,715 |
|  | SD1 | Constant | 72,922 | 1,688 |  | 43,212 | 0,000 |
|  |  | Slope | -0,019 | 0,124 | -0,019 | -0,154 | 0,878 |
|  | SD2 | Constant | 72,915 | 1,163 |  | 62,720 | 0,000 |
|  |  | Slope | -0,006 | 0,023 | -0,031 | -0,258 | 0,797 |
|  | SampEn | Constant | 69,129 | 4,004 |  | 17,266 | 0,000 |
|  |  | Slope | 2,415 | 2,665 | 0,108 | 0,906 | 0,368 |
|  | D2 | Constant | 72,829 | 0,945 |  | 77,107 | 0,000 |
|  |  | Slope | -0,218 | 0,859 | -0,031 | -0,254 | 0,800 |
| HR, min^-1^ | SDNN, ms | Constant | 78,658 | 2,892 |  | 27,194 | 0,000 |
|  |  | Slope | -0,339 | 0,105 | -0,363 | -3,239 | 0,002 |
|  | RMSSD, ms | Constant | 77,520 | 2,120 |  | 36,562 | 0,000 |
|  |  | Slope | -0,453 | 0,112 | -0,439 | -4,057 | 0,000 |
|  | pNN50, % | Constant | 70,717 | 1,150 |  | 61,469 | 0,000 |
|  |  | Slope | -0,370 | 0,200 | -0,218 | -1,851 | 0,068 |
|  | TP, ms^2^ | Constant | 72,329 | 1,479 |  | 48,903 | 0,000 |
|  |  | Slope | -0,004 | 0,002 | -0,271 | -2,337 | 0,022 |
|  | VLF, ms^2^ | Constant | 69,666 | 3,970 |  | 17,550 | 0,000 |
|  |  | Slope | 0,004 | 0,068 | 0,007 | 0,055 | 0,956 |
|  | LF, ms^2^ | Constant | 65,655 | 2,253 |  | 29,145 | 0,000 |
|  |  | Slope | 0,158 | 0,075 | 0,247 | 2,119 | 0,038 |
|  | HF, ms^2^ | Constant | 74,884 | 1,948 |  | 38,437 | 0,000 |
|  |  | Slope | -0,294 | 0,097 | -0,341 | -3,014 | 0,004 |
|  | SD1 | Constant | 77,929 | 2,110 |  | 36,934 | 0,000 |
|  |  | Slope | -0,665 | 0,155 | -0,459 | -4,288 | 0,000 |
|  | SD2 | Constant | 72,190 | 1,594 |  | 45,301 | 0,000 |
|  |  | Slope | -0,061 | 0,031 | -0,227 | -1,936 | 0,057 |
|  | SampEn | Constant | 86,951 | 5,265 |  | 16,516 | 0,000 |
|  |  | Slope | -11,580 | 3,505 | -0,370 | -3,304 | 0,002 |
|  | D2 | Constant | 71,415 | 1,290 |  | 55,340 | 0,000 |
|  |  | Slope | -2,412 | 1,173 | -0,240 | -2,056 | 0,044 |
| 40^th^ minute of DI | | | | | | | |
| SBP, mm Hg | SDNN, ms | Constant | 122,958 | 4,240 |  | 29,000 | 0,000 |
|  |  | Slope | -0,293 | 0,100 | -0,372 | -2,914 | 0,005 |
|  | RMSSD, ms | Constant | 119,539 | 3,372 |  | 35,452 | 0,000 |
|  |  | Slope | -0,299 | 0,110 | -0,351 | -2,727 | 0,009 |
|  | pNN50, % | Constant | 114,001 | 2,137 |  | 53,340 | 0,000 |
|  |  | Slope | -0,350 | 0,172 | -0,270 | -2,040 | 0,046 |
|  | TP, ms^2^ | Constant | 115,072 | 2,547 |  | 45,184 | 0,000 |
|  |  | Slope | -0,002 | 0,001 | -0,252 | -1,896 | 0,063 |
|  | VLF, ms^2^ | Constant | 113,788 | 2,842 |  | 40,034 | 0,000 |
|  |  | Slope | -0,003 | 0,003 | -0,132 | -,972 | 0,335 |
|  | LF, ms^2^ | Constant | 113,752 | 2,181 |  | 52,155 | 0,000 |
|  |  | Slope | -0,003 | 0,002 | -0,232 | -1,736 | 0,088 |
|  | HF, ms^2^ | Constant | 113,896 | 2,213 |  | 51,466 | 0,000 |
|  |  | Slope | -0,007 | 0,004 | -0,236 | -1,768 | 0,083 |
|  | SD1 | Constant | 118,991 | 3,494 |  | 34,052 | 0,000 |
|  |  | Slope | -0,405 | 0,167 | -0,316 | -2,427 | 0,019 |
|  | SD2 | Constant | 123,584 | 4,296 |  | 28,768 | 0,000 |
|  |  | Slope | -0,232 | 0,077 | -0,384 | -3,025 | 0,004 |
|  | SampEn | Constant | 117,480 | 9,847 |  | 11,930 | 0,000 |
|  |  | Slope | -3,863 | 6,471 | -0,082 | -0,597 | 0,553 |
|  | D2 | Constant | 117,520 | 2,593 |  | 45,314 | 0,000 |
|  |  | Slope | -4,158 | 1,374 | -0,384 | -3,027 | 0,004 |
| DBP, mm Hg | SDNN, ms | Constant | 70,479 | 2,341 |  | 30,108 | 0,000 |
|  |  | Slope | -0,076 | 0,055 | -0,186 | -1,376 | 0,175 |
|  | RMSSD, ms | Constant | 69,506 | 1,851 |  | 37,541 | 0,000 |
|  |  | Slope | -0,075 | 0,060 | -0,168 | -1,243 | 0,219 |
|  | pNN50, % | Constant | 68,094 | 1,149 |  | 59,267 | 0,000 |
|  |  | Slope | -0,084 | 0,092 | -0,124 | -0,907 | 0,368 |
|  | TP, ms^2^ | Constant | 68,436 | 1,362 |  | 50,265 | 0,000 |
|  |  | Slope | -0,001 | 0,001 | -0,128 | -0,939 | 0,352 |
|  | VLF, ms^2^ | Constant | 68,256 | 1,490 |  | 45,804 | 0,000 |
|  |  | Slope | -0,001 | 0,002 | -0,087 | -0,634 | 0,529 |
|  | LF, ms^2^ | Constant | 67,950 | 1,165 |  | 58,321 | 0,000 |
|  |  | Slope | -0,001 | 0,001 | -0,088 | -0,643 | 0,523 |
|  | HF, ms^2^ | Constant | 68,047 | 1,182 |  | 57,590 | 0,000 |
|  |  | Slope | -0,002 | 0,002 | -0,104 | -0,759 | 0,451 |
|  | SD1 | Constant | 69,201 | 1,903 |  | 36,362 | 0,000 |
|  |  | Slope | -0,092 | 0,091 | -0,138 | -1,013 | 0,316 |
|  | SD2 | Constant | 70,788 | 2,377 |  | 29,778 | 0,000 |
|  |  | Slope | -0,063 | 0,042 | -0,201 | -1,492 | 0,142 |
|  | SampEn | Constant | 67,394 | 5,154 |  | 13,076 | 0,000 |
|  |  | Slope | 0,102 | 3,387 | 0,004 | 0,030 | 0,976 |
|  | D2 | Constant | 69,803 | 1,404 |  | 49,717 | 0,000 |
|  |  | Slope | -1,615 | 0,744 | -0,286 | -2,171 | 0,034 |
| HR, min^-1^ | SDNN, ms | Constant | 71,218 | 2,628 |  | 27,103 | 0,000 |
|  |  | Slope | -0,164 | 0,062 | -0,341 | -2,641 | 0,011 |
|  | RMSSD, ms | Constant | 67,846 | 2,152 |  | 31,528 | 0,000 |
|  |  | Slope | -0,112 | 0,070 | -,216 | -1,608 | 0,114 |
|  | pNN50, % | Constant | 65,258 | 1,355 |  | 48,151 | 0,000 |
|  |  | Slope | -0,055 | 0,109 | -,069 | -0,502 | 0,618 |
|  | TP, ms^2^ | Constant | 66,310 | 1,587 |  | 41,795 | 0,000 |
|  |  | Slope | -0,001 | 0,001 | -,173 | -1,275 | 0,208 |
|  | VLF, ms^2^ | Constant | 69,208 | 3,445 |  | 20,091 | 0,000 |
|  |  | Slope | -0,087 | 0,066 | -,179 | -1,325 | 0,191 |
|  | LF, ms^2^ | Constant | 59,089 | 2,746 |  | 21,522 | 0,000 |
|  |  | Slope | 0,188 | 0,081 | ,303 | 2,311 | 0,025 |
|  | HF, ms^2^ | Constant | 65,096 | 2,374 |  | 27,426 | 0,000 |
|  |  | Slope | -0,010 | 0,110 | -,013 | -0,094 | 0,925 |
|  | SD1 | Constant | 68,336 | 2,186 |  | 31,255 | 0,000 |
|  |  | Slope | -0,191 | 0,104 | -,244 | -1,830 | 0,073 |
|  | SD2 | Constant | 71,501 | 2,669 |  | 26,791 | 0,000 |
|  |  | Slope | -0,129 | 0,048 | -,348 | -2,706 | 0,009 |
|  | SampEn | Constant | 81,921 | 5,559 |  | 14,738 | 0,000 |
|  |  | Slope | -11,394 | 3,653 | -,394 | -3,120 | 0,003 |
|  | D2 | Constant | 67,416 | 1,654 |  | 40,748 | 0,000 |
|  |  | Slope | -1,800 | 0,876 | -0,272 | -2,054 | 0,045 |
